# Transgenic expression of antimicrobial peptide D2A21 confers resistance to diseases incited by *Pseudomonas syringae* pv. *tabaci* and *Xanthomonas citri*, but not *Candidatus* Liberibacter asiaticus

**DOI:** 10.1371/journal.pone.0186810

**Published:** 2017-10-19

**Authors:** Guixia Hao, Shujian Zhang, Ed Stover

**Affiliations:** U. S. Horticultural Research Laboratory, USDA-ARS, Fort Pierce, FL, United States of America; USDA/ARS, UNITED STATES

## Abstract

Citrus Huanglongbing (HLB) associated with ‘*Candidatus* Liberibacter asiaticus’ (Las) and citrus canker disease incited by *Xanthomonas citri* are the most devastating citrus diseases worldwide. To control citrus HLB and canker disease, we previously screened over forty antimicrobial peptides (AMPs) *in vitro* for their potential application in genetic engineering. D2A21 was one of the most active AMPs against *X*. *citri*, *Agrobacterium tumefaciens* and *Sinorhizobium meliloti* with low hemolysis activity. Therefore, we conducted this work to assess transgenic expression of D2A21 peptide to achieve citrus resistant to canker and HLB. We generated a construct expressing D2A21 and initially transformed tobacco as a model plant. Transgenic tobacco expressing D2A21 was obtained by *Agrobacterium*-mediated transformation. Successful transformation and *D2A21* expression was confirmed by molecular analysis. We evaluated disease development incited by *Pseudomonas syringae* pv. *tabaci* in transgenic tobacco. Transgenic tobacco plants expressing D2A21 showed remarkable disease resistance compared to control plants. Therefore, we performed citrus transformations with the same construct and obtained transgenic Carrizo citrange. Gene integration and gene expression in transgenic plants were determined by PCR and RT-qPCR. Transgenic Carrizo expressing D2A21 showed significant canker resistance while the control plants showed clear canker symptoms following both leaf infiltration and spray inoculation with *X*. *citri* 3213. Transgenic Carrizo plants were challenged for HLB evaluation by grafting with Las infected rough lemon buds. Las titer was determined by qPCR in the leaves and roots of transgenic and control plants. However, our results showed that transgenic plants expressing D2A21 did not significantly reduce Las titer compared to control plants. We demonstrated that transgenic expression of D2A21 conferred resistance to diseases incited by *P*. *syringae* pv. *tabaci* and *X*. *citri* but not Las. Our results underscore the difficulty in controlling HLB compared to other bacterial diseases.

## Introduction

Huanglongbing (HLB) and citrus canker are serious diseases associated with the bacterial pathogens *Candidatus* Liberibacter asiaticus (Las) and *Xanthomonas citri* respectively. The first Las infected tree was reported in Florida in 2005 [1], and HLB has spread to California and Texas as well as most other citrus-growing states. Almost ninety percent of citrus trees are Las-infected in Florida, which has greatly compromised the $9 billion citrus industry. There are no effective strategies to control HLB once trees are infected and no commercial HLB-resistant citrus scion cultivars have been identified. Currently various management strategies such as thermal-therapy, chemotherapy, enhanced nutrient programs and aggressive psyllid management have been studied to reduce disease progress and prolong production in infected trees [2, 3, 4]. Recently, antibacterial compounds were approved to apply on infected citrus trees for HLB management [5].

*X*. *citri* infects all citrus varieties and many citrus relatives, causing the citrus canker disease. The canker disease is contagious and destructive, with sweet orange (*C*. *sinensis* Osb.) and grapefruit (*C*. *paradisi* Macf.) reported to be highly susceptible to canker disease [6]. A citrus gene has been identified that is associated with canker susceptibility, *CsLOB1*, which is a member of the lateral organ boundaries domain gene family. Citrus canker is induced by interaction between *CsLOB1* and TAL (transcriptional activator-like) effectors of *X*. *citri* [7]. In a recent report, it was shown that *CsLOB1* disrupted by CRISPR genome editing enhanced canker resistance in citrus [8]. Due to the difficulty in culturing the Las bacteria, it is more challenging to investigate and manipulate HLB pathogenesis for disease resistance.

Antimicrobial peptides (AMPs) have been widely studied for producing transgenic plants resistant to microbial diseases. More than nine hundred natural and synthetic AMPs have been reported. AMPs are short sequence peptides with broad spectrum antimicrobial activity against bacteria and fungi and are produced by most eukaryotic organisms. Most AMPs damage pathogen cells by inhibiting chitin synthase or β-D-glucan synthase, followed by pore forming and bacterial membrane disruption [9, 10]. In addition, AMPs are also reported to interfere with cell division, macromolecular synthesis, and cell wall formation [11]. Plants have two levels of active defense responses against pathogens: pathogen-associated molecular patterns (PAMP)-triggered immunity (PTI) and effector-triggered immunity (ETI) [12]. In plants AMPs act as the first line of defense against invading plant pathogens, often with some basal level of expression, but further induced as part of active defense [13]. In addition to direct activity against microbes, AMPs also contribute to plant immunity by activating defense responses associated with PTI/ETI including ROS production and MAPK signaling, and interacting with other AMPs and pathways involving hormonal cross-talk and sugar signaling [14]. Transgenic plants expressing some AMPs have shown promising results, displaying enhanced disease resistance [15]. Compared to naturally produced peptides, synthetic peptides have shown rapid biocontrol or biostatic ability against various fungal and bacterial pathogens at low concentrations and can be designed to reduce toxicity to mammalian cells [16]. In addition, synthetic peptides are often designed to resist degradation by fungal and plant proteases and show target specificity and increased efficacy [15]. Synthetic peptide D2A21 was one of the most active AMPs and also had low hemolytic activity in our in vitro screening of 40 AMPs against *X*. *citri*, *S*. *meliloti* and *A*. *tumefaciens* [17]. In addition, it has been demonstrated that D2A21 has broad-spectrum antifungal activity in vitro against various tree pathogens including *Gremmeniella abietina*, *Ophiostoma ulmi*, *Cylindrocladium floridanum* and *Cronartium ribicola* [18].

Conventional breeding for disease-resistant crop varieties is often a long-term endeavor. It is especially challenging for perennial tree crops such as citrus with long juvenile periods and complex genetic backgrounds, resulting in many years between generations. Furthermore the HLB-resistance which has been identified in the citrus gene pool is in distant relatives, requiring numerous generations to introgress this resistance into market phenotypes. Citrus transgenic plants expressing various genes, including AMPs and host defense signaling components, have been shown to enhance canker resistance [19–24]. However, only a few genetic engineering studies have resulted in relative HLB resistance as expressed by reducing Las titer [25–27]. It is noteworthy that transgenic citrus plants expressing spinach defensins were reported to be highly resistant to HLB [28]. The aim of our research is to achieve HLB and canker disease resistance by transforming commercial varieties of citrus with highly effective AMPs. As described above, screening results showed that D2A21 is one of the most effective peptides against several bacterial species [17]. Therefore, the sequence coding D2A21 was synthesized and cloned into binary vector pBinARS/Plus for plant transformation. We transformed and obtained transgenic tobacco and citrus expressing D2A21. We showed that the transgenic tobacco expressing D2A21 enhanced resistance to disease incited by *P*. *tabaci*. We demonstrated that transgenic citrus expressing D2A21 significantly reduced canker development caused by *X*. *citri*. However, nine months after graft inoculation, Las titer was not significantly reduced in transgenic plant expressing D2A21 compared to control plants.

## Materials and methods

### Phytotoxicity of D2A21 peptide and its inhibition efficiency against Liberibacter crescens (Lcr)

Four-week old plants of *N*. *benthamiana* and *N*. *tabacum* cv. Samsun were used for phytotoxicity assays along with small potted plants of Hamlin sweet orange. Synthesized D2A21 (provided by AgroMed LLC, Annapolis, MD) was inoculated into fully expanded plant leaves at concentrations of 10, 50, 100, 150 or 200 μM using a needleless syringe after pricking with a needle. Six independent inoculations were carried out in a single leaf, and three leaves from different plants were inoculated. For *N*. *benthamiana*, photographs were taken overnight after inoculation, because the phytotoxic response was very rapid. For *N*. *tabacum*, cv. Samsun and citrus plants, photographs were taken three days after inoculation.

Liberibacter crescens strain BT-1 was grown in liquid BM7 medium: 2 g of alpha-ketoglutarate, 10 g of N-(2-acetamido)-2-aminoethanesulfonic acid (ACES) buffer, and 3.75 g of KOH in 550 ml distilled water; pH then was adjusted to 6.9 and autoclaved for 20 min, followed by the addition of 150 ml of filter-sterilized fetal bovine serum (HyClone Laboratories, Logan, UT, USA) and 300 ml of modified Grace's insect culture medium (TNM-FH; HyClone Laboratories) when cool to room temperature [29]. In vitro inhibition tested were conducted in 96-well plates with 3 days old Lcr culture (OD600 = 0.25) diluted to OD600 = 0.02. Diluted Lcr (145 μl/well) was seeded onto polystyrene 96-well plates. 5μl D2A21 solution with varying concentrations or sterilized distilled water were added into each well to reach the final concentrations of 40, 20, 10, 5, 2.5, 1.2, 0.6, 0.3, 0.15, 0.075 and 0 μM. Three replicates were included at each concentration and three wells with 200 ul BM7 medium were used as controls in each plate. Plates were briefly centrifuged and then incubated at 28°C with shaking at 180 rpm. Bacterial growth was measured (OD_600_) at 0h, 22h, 28h, 42h, and 48h after inoculation. The Minimum inhibitory concentration (MIC) value was determined by examining plots of growth data and identifying the lowest concentration where there was no increase in OD600 between 0 and 48 h. The experiments were performed four times.

### Synthesis of D2A21 coding sequence and binary vector construction

**Fig 1:**
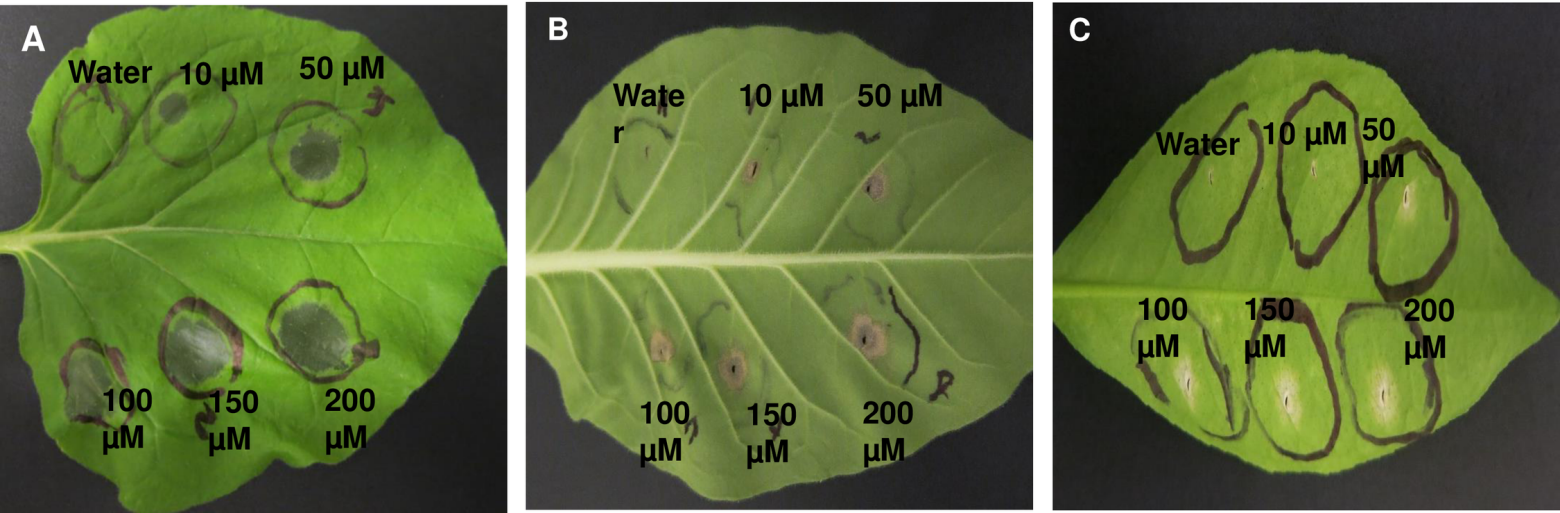
Phytotoxicity tests using chemically synthesized D2A21. Leaves of *N*. *benthamiana* **(A)**, tobacco **(B)** and citrus **(C)** were infiltrated with a concentration range of D2A21 with water infiltration alone used as a control. Photographs were taken after 14 hr (**A**) or three days after infiltration (**B** and **C**). Three leaves from each of three independent plants were infiltrated and the experiments were repeated three times with similar results.

D2A21 contains 23 amino acids. DNA sequence encoding the peptide D2A21 was synthesized by DNA2.0 (Fig 1). The gene was obtained by digestion of the vector pJ224 with *Sma* I and *kpn* I and then ligated to binary vector pBinARS/Plus driven by the double 35S (D35S) promoter (Fig 1). The ligation product was used to transform *E*. *coli* TOPO10 competent cells (Invitrogen, CA). The positive clones were identified by single colony PCR, followed by plasmid isolation and sequencing to confirm the consensus clone. The plasmids carrying the sequence for D2A21 were introduced into *A*. *tumefaciens* EHA105 by electroporation. The binary vector pBinARS/Plus carrying GUS (*β-D-glucuronidase*) driven by the D35S promoter was also introduced into *A*. *tumefaciens* EHA105 as a control.

### Tobacco and citrus transformation

Transformation of tobacco (*N*. *tabacum*, cv. Samsun) was performed using *Agrobacterium*-mediated leaf disk transformation [30]. Leaf disks were cultured on Murashige and Skoog (MS) medium with addition of 1 mg/L 6-benzylaminopurine, 0.1 mg/L NAA, 150 mg/L kanamycin and 200mg/L cefotaxime. The kanamycin (kan) resistant plants were selected and rooted in MS medium containing 0.1 mg/L NAA, 100 mg/L kan and 100mg/L cefotaxime. Transformation of citrus was performed using *Agrobacterium*-mediated transformation as described [25]. The rooted plants were potted in a commercial soil-less mix for further tests in a controlled green house.

### Molecular analysis of transgenic plants

DNA was isolated from transgenic tobacco and citrus leaves using the DNeasy plant kit (Qiagen, Valencia, CA). Primers were designed to span the fragment from the D35S promoter region to the nos terminator region. The D35S promoter primer: 5’-GACGCACAATCCCACTATCC -3’ and the nos terminator primer: 5’-TTTGCGCGCTATATTTTGTTT -3’ were used to amplify plant genomic DNA using Taq polymerase (Epicenter, Madison, WI). The PCR reaction was as follows: denature at 95°C for 3 min followed by 32 cycles of 95°C for 30 s to denature, 53°C for 30 s to anneal and 72°C 1 min for extension. The PCR products were run on a 1% agarose gel and were stained with ethidium bromide.

RNA was extracted from transgenic tobacco and citrus leaves using Trizol reagent (Sigma-Aldrich, St. Louis, MO). Total RNA was quantified using the Nanodrop Spectrophotometer (Thermo Scientific, Wilmington, DE) and then treated with RQ1 RNase-free DNase from Promega Corp (Madison, WI). In a 20 μL reaction, 1.5 μg DNase-treated RNA was used for first-strand cDNA synthesis with 0.5 μg of oligo (dT) primer and 1 μL of SuperScript® III reverse transcriptase (Thermo Fisher Scientific, Waltham, MA). The absence of genomic DNA contamination was verified using a negative control without the reverse transcriptase. Semi-quantitative PCR was performed as described above with cDNA as template using 26 cycles for transgenic lines carrying *D2A21*.

RT-qPCR was set up with three technical replicates using Bright Green (Sigma-Aldrich, St. Louis, MO). In 25 μL amplification reactions, the following materials were added: 12.5 μL of Bright Green PCR Master Mix system, 0.5 μL of 250 nM forward and reverse primers, and 2.0 μL of 1:10 diluted cDNA template. The qPCR was run in an ABI7500 thermal cycler (Thermo Fisher Scientific): 95°C for 2 min, 40 cycles with 30 s for denaturation at 95°C and 30 s for extension at 60°C. Primers for *D2A21* were 5’- AAGTTCGCTAAGAAATTTGCCA-3’ and 5’- TGTATAATTGCGGGACTCTAATCA-3’. For tobacco plants, the elongation factor 1 alpha (*NtEF1α*) from *N*. *tabacum* was amplified and used to normalize the values as an internal control with primers 5′-GACCACTGAAGTTGGATCTGTTG-3’ and 5’-TAGCACCAGTTGGGTCCTTCTT-3’. For citrus, the Glyceraldehyde-3-phosphate dehydrogenase C2 (*GAPC2*) was amplified and used to normalize the values as an internal control with primers 5’-TCTTGCCTGCTTTGAATGGA-3’ and 5’-TGTGAGGTCAACCACTGCGACAT -3’. To compare the transcript levels, a ratio of relative gene expression was calculated from the 2^ΔCt^ values of a sample versus non transformed control plant which was set at a Ct value of 40 for each target gene. ΔCt = Ct (internal control)—Ct (target gene). The qPCR was performed twice for statistical analysis. Gene expression s were analyzed by t test, with* indicating significant at p value <0.05.

### Pathogenesis assays

#### *P*. *syringae* pv. *tabaci* challenge on tobacco

*P*. *syringae* pv. *tabaci* strain was obtained from Dr. Tim Schubert at the Florida Department of Agriculture. Overnight cultures of *P*. *syringae* pv. *tabaci* strain were centrifuged and diluted to a range of concentrations in sterile distilled water. Dilutions of 10^2^, 10^3^, 10^4^, 10^5^ and 10^6^ CFU/ml were verified by the standard plate-dilution method. Three leaves of each plant were inoculated with each bacterial inoculum level. The bacterial suspensions were infiltrated from the abaxial side into leaves of transgenic tobacco plants expressing D2A21 and GUS transformed controls using a syringe. Inoculated plants were incubated for 14 days and then disease development was evaluated and photographed. Number of lesions was counted and compared between transgenic tobacco and GUS-expressing plants with inoculation concentration at 10^4^ CFU/ml. The inoculation experiment was repeated twice with similar results. The data are means from six infiltration zones from three leaves 14 days after inoculation. Bars represent standard deviations from six inoculated zones. * indicates a significant difference at p value < 0.05.

#### Citrus canker challenge with *X*. *citri*

Canker pathogenesis assay was performed as described [21]. Briefly, overnight cultures of *X*. *citri* were centrifuged and diluted to 10^6^ CFU/ml in sterile distilled water. The bacterial suspensions were infiltrated from the abaxial side into leaves of citrus plants expressing D2A21 and control plants, using a needleless syringe after pricking with a needle. Inoculated plants were incubated for two weeks and then canker symptoms were scored and photographed. Additional plants were spray-inoculated with a 10^6^ CFU/mL suspension of *X*. *citri* with addition of 0.02% silwet-77. Several young fully expanded leaves on each plant were spray-inoculated. Disease development was evaluated frequently after inoculation. Photographs were taken three weeks after spray inoculation. Three independent experiments were performed with similar results.

#### HLB challenge via graft inoculation

Transgenic Carrizo plants expressing D2A21 and nontransgenic controls were propagated by root cuttings. Because Carrizo is used for rootstock, graft transmission was chosen for HLB challenge. Transgenic plants for graft inoculation were selected according to D2A21 expression level determined by RT-qPCR. The experiments were performed by propagating eight replications for each transgenic plant and controls. The budwood used for inoculation was rough lemon infected with Las. About 2–3 inches from the top of each propagated plant was removed and grafted with two HLB-infected rough lemon buds. Las titers in these buds were determined to have Ct values ranging from 24.0 to 26.0 by qPCR. Eight wildtype Carrizo (CK) and D2A21 transgenic Carrizo plants from nine independent transgenic events were used for graft inoculation with infected rough lemon buds. The following successful grafts were obtained: CK (six plants), C16 (three plants), C18 (six plants), C19 (three plants), C20 (five plants), C21 (three plants), C22 (three plants), C24 (three plants), C25 (one plant) and C26 (five plants). Graft inoculated plants were grown in the greenhouse maintaining the rough lemon as the scion. Symptoms were evaluated periodically. Symptomatic and asymptomatic leaves from untransformed rough lemons scion and fibrous roots from transgenic and control rootstocks were sampled at nine months post graft inoculation. Leaf midribs were chopped and DNA isolated using the DNeasy plant kit (Qiagen, Valencia, CA). Root DNA was extracted using the PowerSoil DNA Isolation Kit (Mo Bio Laboratories, Qiagen) [31]. Las titer was determined using an ABI7500 thermal cycler (Thermo Fisher Scientific, Waltham, MA) as described above. Las long primers were used for Las detection and citrus dehydrogenase (CD) was used as the internal control to examine DNA quality. The qPCR reactions were performed with three technical replicates and repeated twice for statistical analysis. Bars represent the means of Ct values from three to six propagated plants from the same parent transgenic plants. Las titer was compared between lines for each tissue type using Kruskal Wallis (p<0.05) and SAS software (SAS Institute Inc., Cary, NC). D2A21-24 was excluded from statistical analysis because few samples were obtained: two samples from old leaves and roots respectively, one sample from a young leaf.

## Results

### Toxicity of D2A21 peptide on plants and Lcr inhibition efficiency

**Fig 2:**
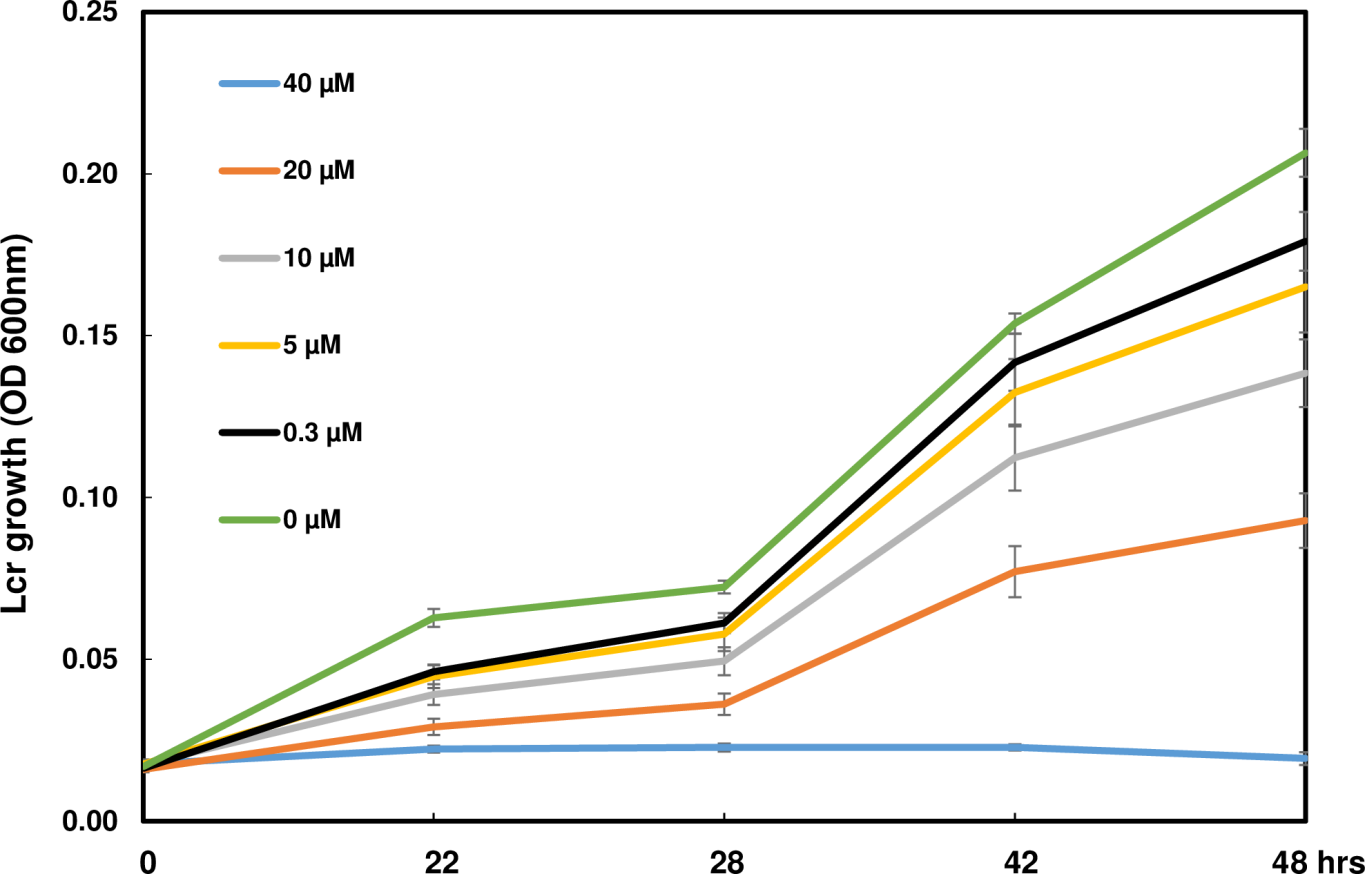
Growth of *Liberibacter crescens (*Lcr*)* in BM7 broth with addition of D2A21. Lcr growth was measured (OD_600_) at 0h, 22h, 28h, 42h, 48h after inoculation with various concentrations of D2A21. Each time point is average of four biological replicates and error bars represent standard error. Growth plots at concentration of D2A21 at 1.2, 0.6, 0.15 and 0.075 μM weren’t shown in the figure for the purpose of easy visualization.

We tested the potential toxicity of D2A21 peptide to plant cells by inoculation of the synthetic peptide into leaves of *N*. *benthamiana*, *N*. *tabacum* and citrus. Cell death was observed in the inoculated leaves with concentration of 10 to 200μM in a dose dependent manner (Fig 1). *N*. *benthamiana* was the most sensitive to D2A21. Cell death was observed at all inoculated concentrations overnight. Compared to *N*. *benthamiana*, *N*. *tabacum* and citrus are more tolerant to D2A21. Cell death was only observed with inoculated concentrations higher than 50μM. This suggests that D2A21 is toxic to *N*. *tabacum* and citrus cells when applied at high concentration. Our earlier study showed that D2A21 can kill bacteria at 1μM [17]. Because Las cannot yet be cultured, we tested D2A21 activity against Lcr, the closely related culturable species to Las [32]. As shown in Fig 2, D2A21 can kill Lcr at 40 μM, which is much higher compared to the concentration to kill Xcc and other tested bacteria [17]. The MIC could be between 20–40 μM, apparently much higher than that against Xcc, but still below the toxic concentration on plants. Together these suggest that *N*. *tabacum* and citrus transgenics expressing D2A21 may permit bacterial disease control without damaging the plants.

### Synthesis and cloning of peptide gene

**Fig 3:**
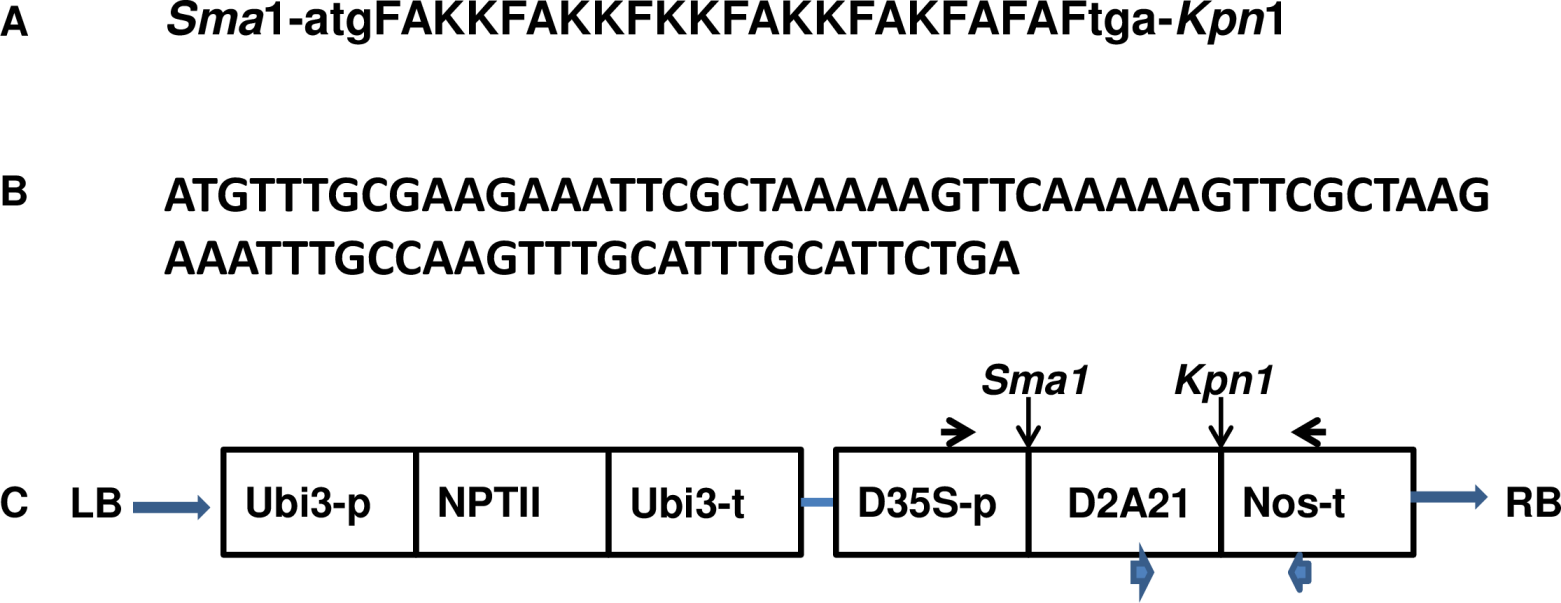
Sequences and diagrams for *D2A21* insertion into binary vector pBinARS/Plus. **A:** Amino acid sequence of D2A21 flanked by restriction sites. **B:** D2A21 nucleotide sequence containing start and stop codons. **C**: Construct components. In addition to *D2A21* gene of interest and *NptII* marker genes: Ubi3-p is ubiquitin promoter; Ubi3-t is ubiquitin terminator; D35S-p is double CaMV 35S promoter; Nos-t is nos terminator; LB is *Agrobacterium* left border; and RB is *Agrobacterium* right border. Arrows: primers for PCR to confirm gene integration located in D35S promoter and nos terminator regions; Arrow heads: primers for RT-PCR and RT-qPCR located in target gene and nos terminator regions. Abbreviations used in the amino acid sequences: A, alanine; F, phenylalanine; K, lysine.

A DNA sequence encoding the D2A21 peptide was synthesized with additional nucleotides to generate digestion sites for cloning with enzymes *Sma* 1 and *Spe* 1 (Fig 3A and 3B). The DNA coding sequences for D2A21 were optimized for efficient expression in dicot plants by DNA2.0. The synthesized gene of D2A21 was cloned into the binary vector pBinARS/Plus and its expression was controlled by the double 35S (D35S) promoter (Fig 3C).

### Gene integration and expression in transgenic *N*. *tabacum*

**Fig 4:**
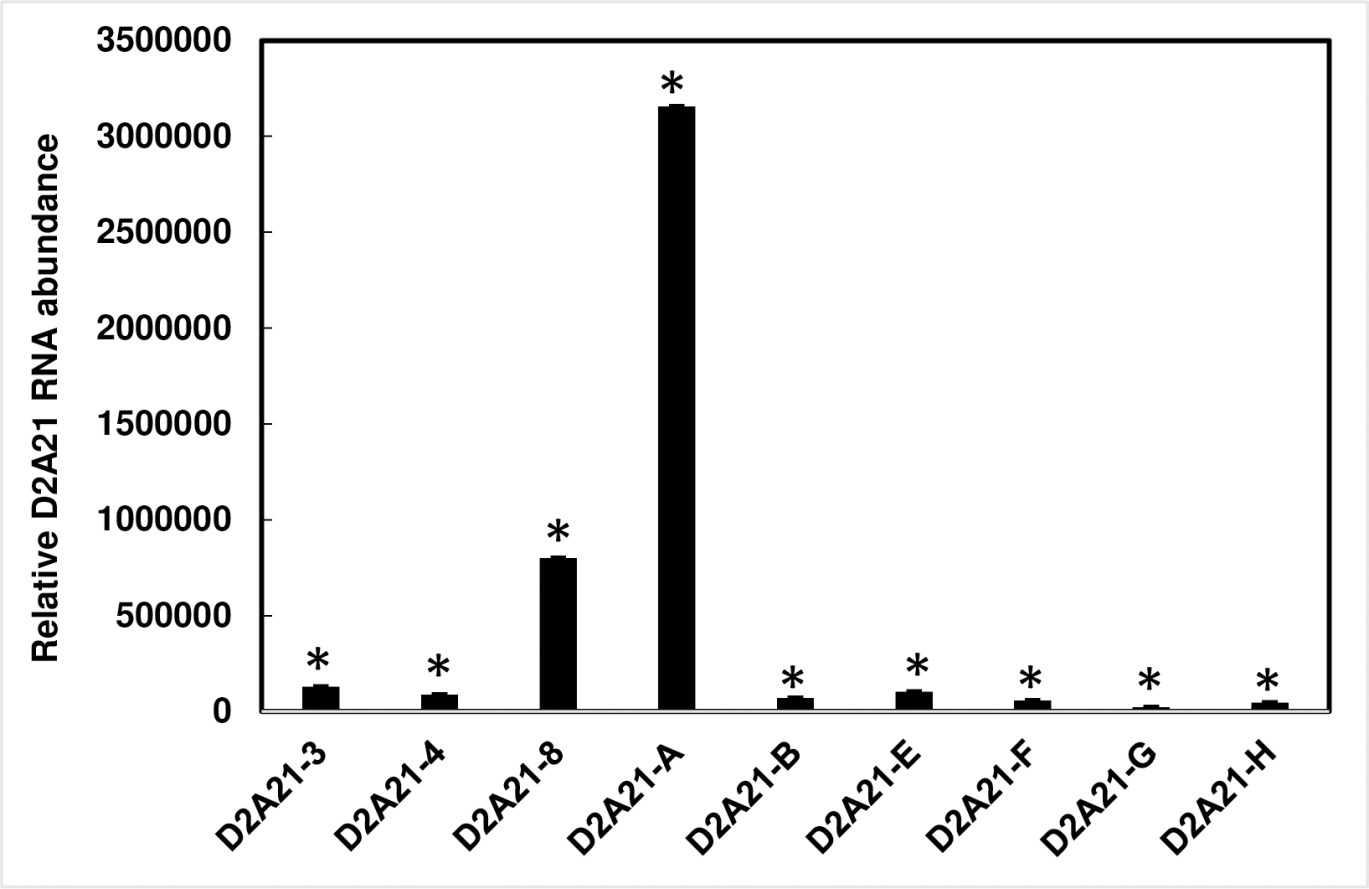
Quantitation analysis of RNA in transgenic tobacco by RT-qPCR. Relative gene expression of *D2A21* in transgenic tobacco was normalized to the expression of the tobacco *EF1α*. Expression fold change is relative to the Gus-expressing control plant, whose Ct value was set as 40 for *D2A21*.

Transgenic *N*. *tabacum* plants carrying the *D2A21* construct were obtained through kanamycin resistance selection conferred by the *NptII* gene. Nine independent kanamycin resistant transgenic plants were generated carrying *D2A21*. Integration of *D2A21* was confirmed by PCR amplification. The expected 350-bp fragment was observed in transgenic plants while a 2,000-bp band reflecting the *gus* gene was present in GUS transformed control plants (Panel A in S1 Fig). With semi-quantitative RT-PCR, all transgenic tobacco plants containing D2A21 showed *D2A21* gene expression (Panel B in S1 Fig). To further compare the gene expression level in these transgenic plants, RT-qPCR was used to determined RNA abundance relative to the internal control gene tobacco *EF1ɑ*. Transgenic line D2A21-A had the highest level of *D2A21* gene expression, followed by D2A21-8 (Fig 4).

### Gene integration and expression in transgenic citrus

**Fig 5:**
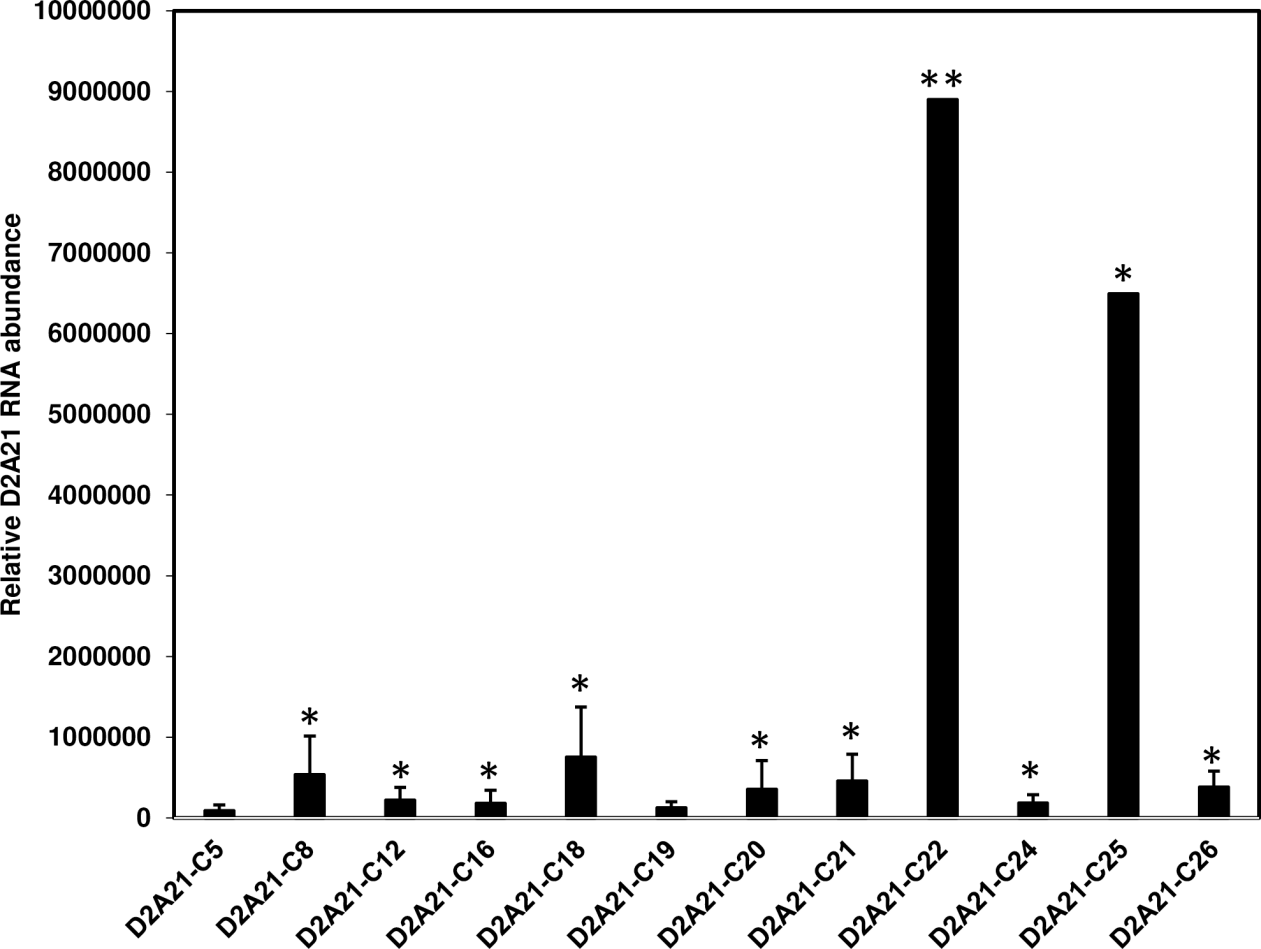
Quantitation analysis of RNA in transgenic citrus by RT-qPCR. Relative gene expression of *D2A21* in transgenic citrus was normalized to the expression of the citrus *GAPC2* gene. Expression fold change is relative to the non-transformed citrus, whose Ct value was set as 40 for *D2A21*.

A total of twenty eight Carrizo transgenic plants were confirmed by PCR amplification to carry the *D2A21* gene (S2 Fig). Twelve transgenic plants carrying the *D2A21* insertion were further analyzed for gene expression. The expression of *D2A21* was confirmed by conventional reverse transcriptase PCR (RT-PCR) in analyzed plants. The *D2A21* transcripts were compared by RT-qPCR and gene expression levels varied greatly among the transgenic lines. In particular, transgenic plant D2A21-C22 displayed the highest *D2A21* transcription levels while transgenic plant D2A21-C25 had an intermediate level of expression. Transgenic plants C8, C18, C21 and C26 showed relatively high transcription levels compared to the remaining transgenic plants (Fig 5).

### Transgenic tobacco expressing *D2A21* displayed disease resistance

**Fig 6:**
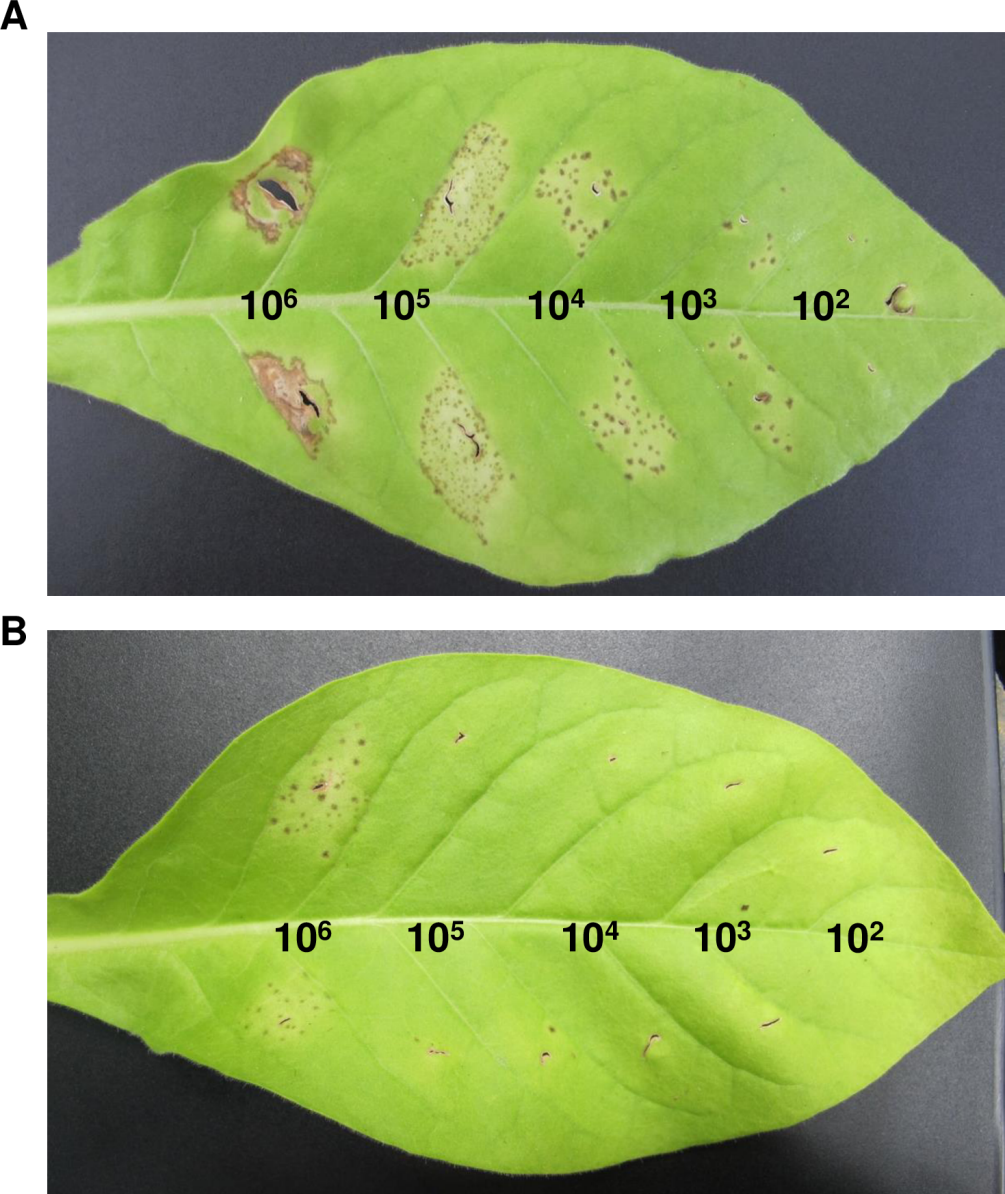
Disease symptoms incited by *P*. *syringae* pv. *tabaci* on leaves of transgenic tobacco lines. **A:** GUS transgenic control; **B:** transgenic plant expressing *D2A21*. The tobacco leaves were infiltrated with *P*. *syringae* pv. *tabaci* at the concentrations of 10^2^, 10^3^, 10^4^,10^5^ and 10^6^ CFU/ml. Photographs were taken 14 days after infiltration.

**Fig 7:**
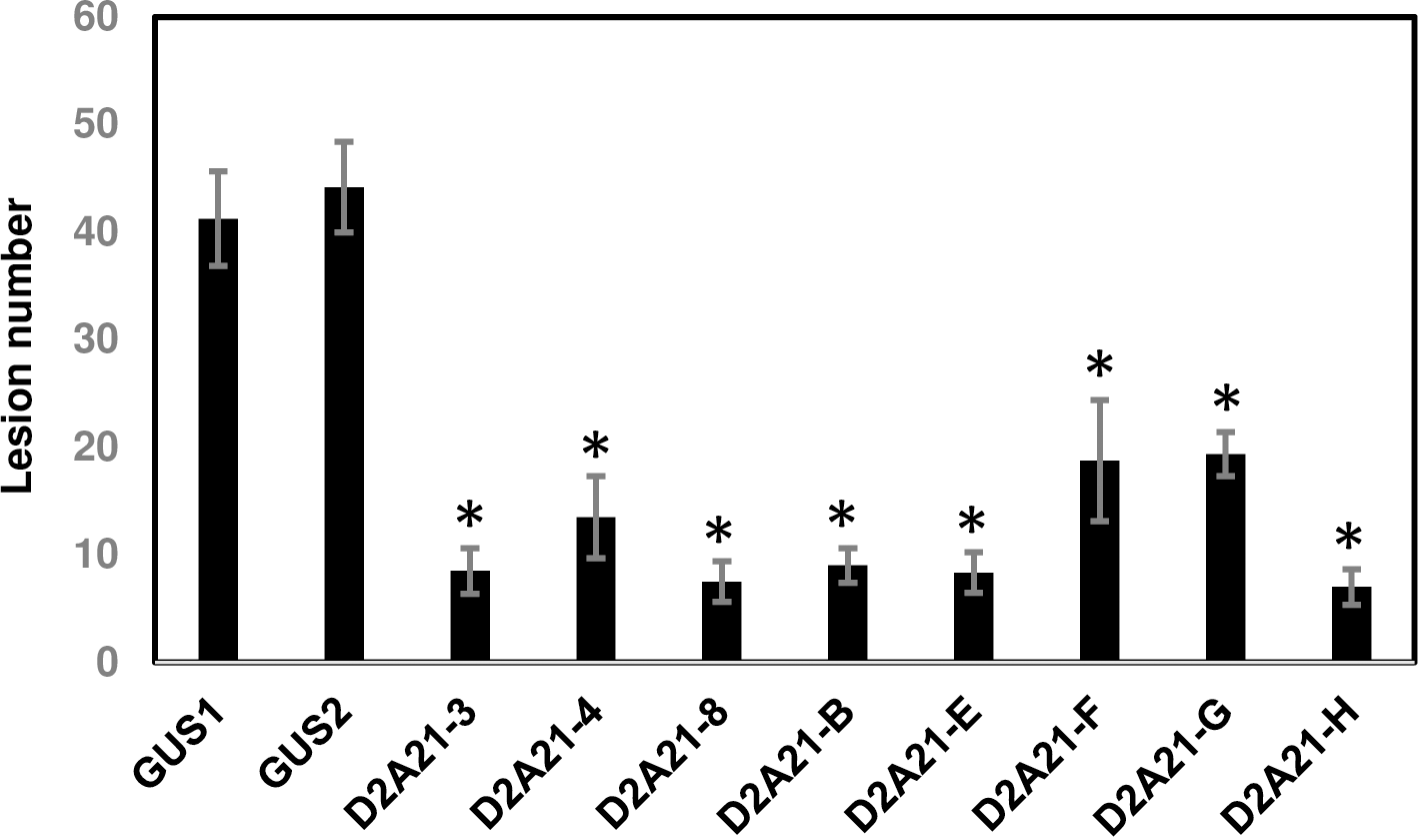
Comparison of lesions in leaves of transgenic tobacco plants expressing *D2A21* and control plant expressing *Gus*. The *P*. *syringae* pv. *tabaci* inoculation concentration was 10^4^ CFU/ml. The lesion numbers were counted from six infiltration zones of three leaves 14 days after inoculation. Bars represent standard deviations from six inoculated zones.

Leaves of different transgenic tobacco lines were infiltrated with *P*. *syringae* pv. *tabaci* at various concentrations to evaluate whether the expression of *D2A21* in transgenic plants can reduce infection. Yellowing or necrotic lesions were first observed 6 day post infiltration (dpi). Symptoms continued to develop through the 14-day evaluation period. On 14 dpi, the GUS transformed control plants showed brown necrosis and cell death at the concentration of 10^6^ CFU/ml and yellowing or brown lesion spots at 10^2^−10^5^ CFU/ml (Fig 6A). Transgenic plants expressing *D2A21* showed remarkable resistance with lesions observed only at the highest infiltration level of 10^6^ CFU/ml, which were comparable to lesions in the control at two orders of magnitude lower CFU (equal to 10^4^ CFU/ml). As shown in Fig 6, transgenic line D2A21-8 only showed some lesion spots in the infiltrated zone with infiltration at 10^6^ CFU/ml while no or few small necrotic lesions were observed with infiltration at 10^2^−10^5^ CFU/ml. Unfortunately, transgenic plant D2A21-A died before further disease challenge, which may be due to toxicity from high expression of *D2A21*. Since it is difficult to count lesion numbers for control plants at high inoculated concentrations, the inoculation of 10^4^ CFU/ml was used to compare lesion number in transgenic tobacco and the control plants. All tested transgenic tobacco expressing D2A21 showed significantly less disease compare to Gus-expressing control plant (Fig 7). Taken together, our results demonstrated that transgenic tobacco expressing *D2A21* reduced infection caused by *P*. *syringae* pv. *tabaci*.

### Transgenic Carrizo expressing *D2A21* displayed canker resistance

**Fig 8:**
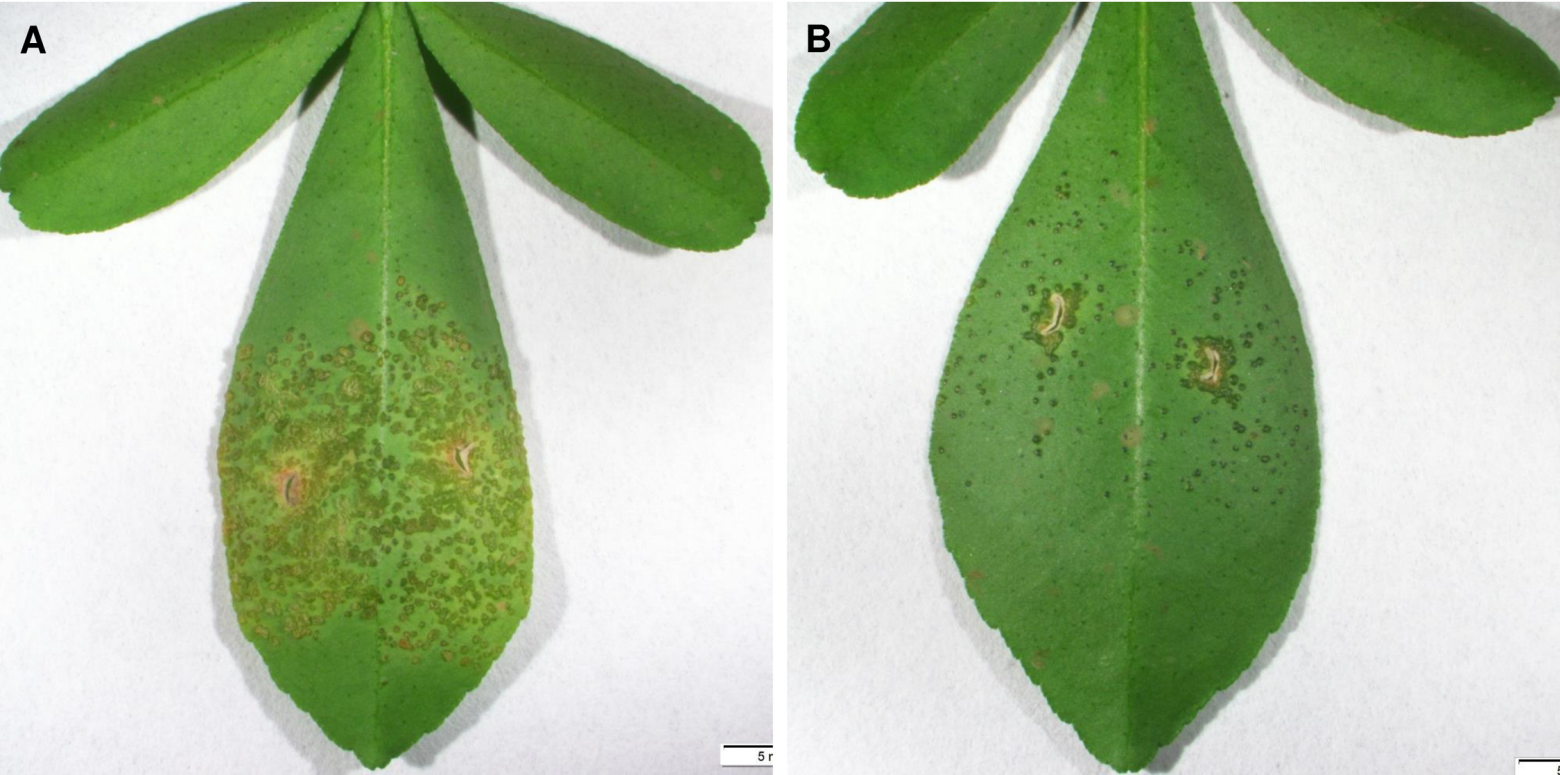
Disease symptoms incited by *X*. *citri* on leaves of Carrizo plants by infiltration assay. **A:** Control plant; **B:** Transgenic plant D2A21-C8 expressing *D2A21*. The citrus leaves were infiltrated with *X*. *citri* at the concentration of 10^6^ CFU/ml. Photographs were taken 10 days after infiltration.

**Fig 9:**
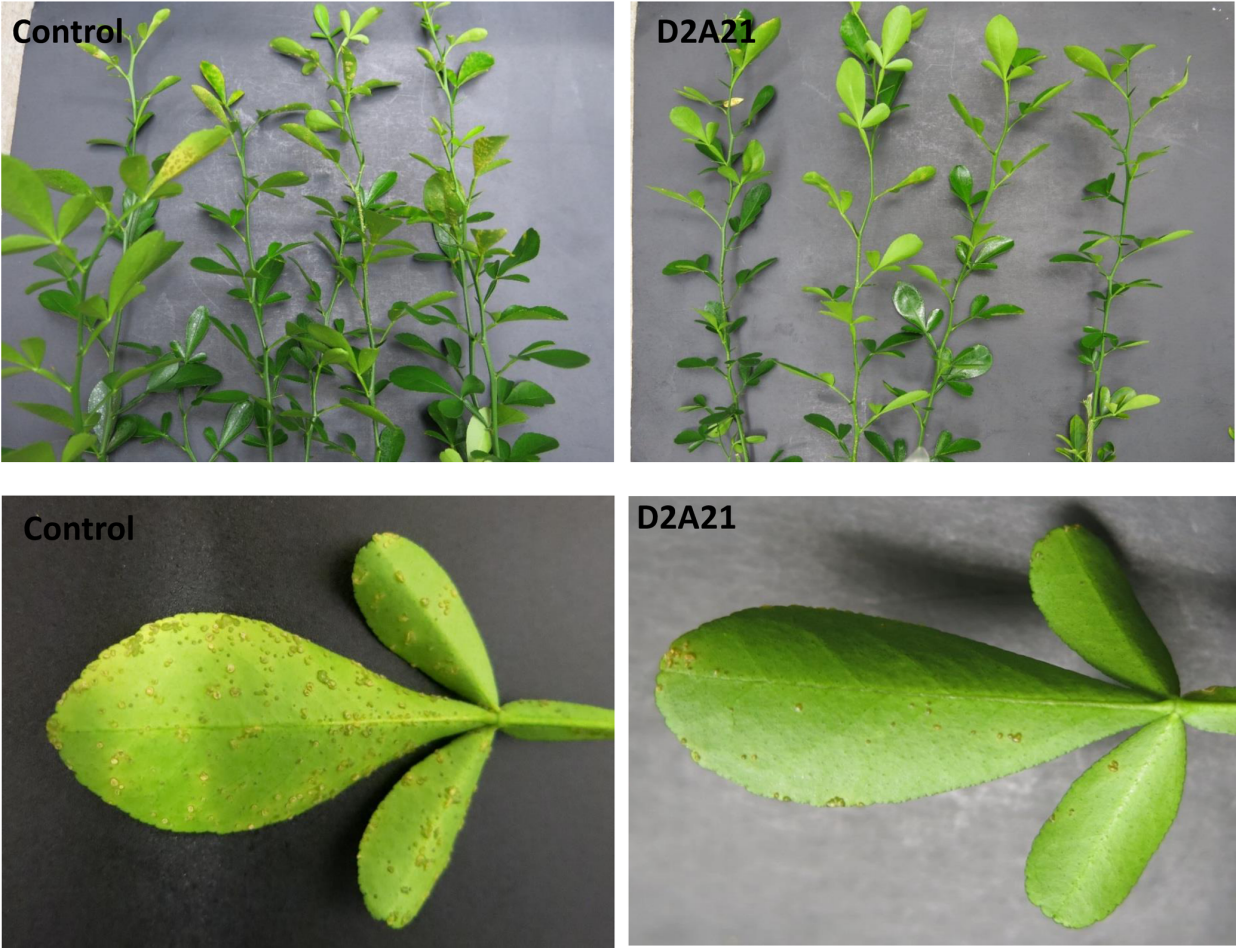
Disease symptoms incited by *X*. *citri* on leaves of transgenic Carrizo plants by spray inoculation. **A:** Control plants; **B:** Transgenic plants expressing *D2A21*. The citrus plants were sprayed with *X*. *citri* at the concentration of 10^6^ CFU/ml. Photographs were taken 14 days after spray inoculation.

To determine whether the *D2A21* transgene conferred resistance to *X*. *citri*, transgenic and control Carrizo plants were inoculated and evaluated. Several regenerated plants confirmed by PCR to be free of transgene insertion were included as controls. The effectiveness of the transgene in reducing disease development was substantial following infiltration at the concentration of 10^6^ CFU/ml (Fig 8). Furthermore, with spray inoculation at 10^6^ CFU/ml, transgenic plants expressing *D2A21* showed marked disease resistance four weeks after inoculation (Fig 9). Most of the tested transgenic Carrizo expressing *D2A21* showed canker resistance compared to control plants following spray inoculation. Taken together, our assays using infiltration and spray inoculation indicated canker resistance in transgenic Carrizo expressing *D2A21*.

### Transgenic Carrizo expressing *D2A21* did not reduce Las titer

**Fig 10:**
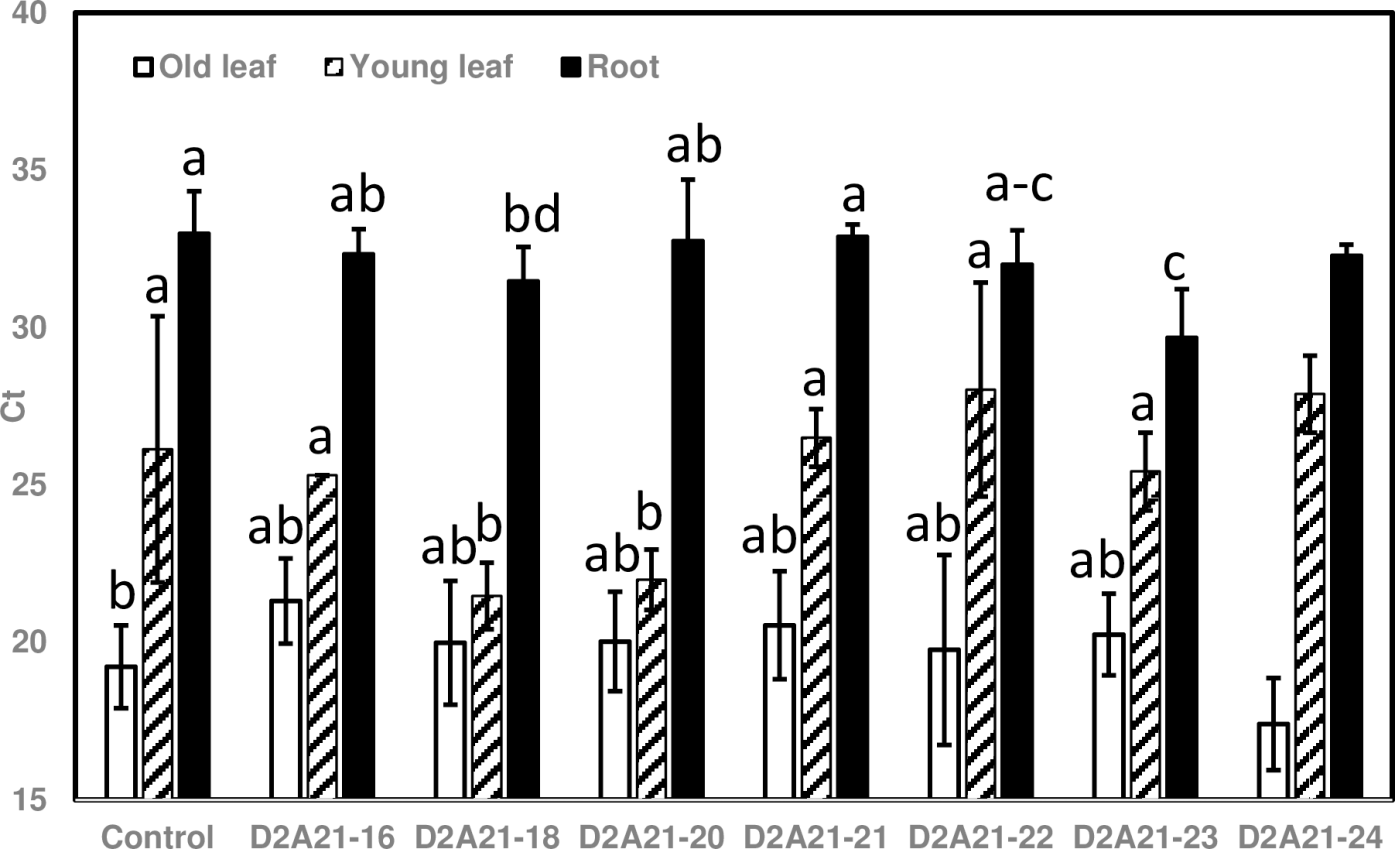
Quantification of Las titer from old leaves, new leaves and roots of the plants expressing *D2A21* in roots vs. control rootstock plants nine months after grafting with highly infected rough lemon scions. CK: nontransformed wild type; D2A21 transgenic plants: C16, C18, C20, C21, C22, C23 and C24. The qPCR reactions were set up in triplicate and repeated twice with similar results. Bars represent Ct values obtained by qPCR so lower values indicate higher Las titer. Results are the means of Ct value from three to six replicated plants. Bars marked with the same letters indicate not significantly different at the p< 0.05 level by Kruskal Wallis statistical analyses.

Typical HLB symptoms including leaf blotchy mottle were observed in rough lemon scions nine month after grafting onto transgenic and control Carrizo. To test whether D2A21 can inhibit Las growth in the roots of transgenic rootstocks, DNA was isolated from fibrous roots of transgenic and control Carrizo. It is evidenced that certain transcription factors, RNAs, peptides and protein can be translocated from rootstock to scion via crossing the graft union [33]. To determine if D2A21 can reduce Las titer in untransformed infected rough lemon scion, DNA was also isolated from old leaves (most with HLB symptoms) and young but fully expanded leaves (without HLB symptoms). Las titer was obtained with Las specific primers (lower Ct indicates higher bacterial titer). Ct values from individual control and transgenic plants were compared (S1 Table). Both in old and young leaves, Las was detected in grafted rough lemon on transgenic plants and control plants. Old rough lemon leaves had higher bacterial titer than young leaves as reflected by lower Ct values in older leaves (Fig 10). Both transgenic and control roots had higher Ct values than in leaves, indicating lower Las populations in the roots. However, no significant difference was observed between wild-type controls and transgenic plants by statistical analysis (Fig 10). Our results indicated that transgenic citrus expressing *D2A21* did not significantly reduce Las titer.

## Discussion

Transformation of crop plants to express AMPs has been an effective approach against some plant diseases. *In vitro* tests of forty synthetic AMPs (against *X*. *citri*, *S*. *meliloti* and *A*. *tumefaciens*) showed that D2A21 was one of the most active AMPs and also had low hemolytic activity [17]. D2A21 has been reported to have broad-spectrum antifungal activity [18]. In this study, we showed that D2A21 has high phytotoxicity to leaves of *N*. *benthamiana* but relatively low phytotoxicity to leaves of *N*. *tabacum* and citrus. *In vitro* testing of D2A21 against Liberibacter crescens, a culturable species closely related to Las, showed that D2A21 was less effective against Liberibacter. We overexpressed *D2A21* in transformed tobacco and citrus. All the transgenic plants appeared normal in growth and phenotype. We verified gene integration and expression in the transgenic plants. We demonstrated that transgenic tobacco and citrus expressing *D2A21* had substantial resistance to disease incited by *P*. *syringae* pv. *tabaci* and *X*. *citri* respectively. However, our HLB challenge assay showed that Las titer was not significantly reduced in transgenic citrus compared to controls plants.

We generated nine independent transgenic tobacco lines expressing *D2A21*. Although no abnormal morphology was observed, the highest expressing transgenic plant (D2A21-A) died before disease challenge possibly due to D2A21 toxicity. It was reported that D2A21 has low pollen toxicity in *Pinus*, *Picea* and *Populus* [34], however we noticed strong toxicity in infiltrated zones of *N*. *benthamiana* leaves (Fig 1). We obtained twenty eight independent transgenic Carrizo and all of them were free of abnormal phenotype and dieback. As in most reports, variation in gene expression was observed in different transgenic lines, likely due to factors such as gene integration site, promoter methylation or gene silencing [35].

The transgenic tobacco plants were shown to inhibit disease development incited by *P*. *syringae* pv. *tabaci* at all inoculation levels from 10^2^ to 10^6^ CFU/ml. No or few lesions were observed at inoculum concentration of 10^2^ to 10^5^ CFU/ml in plants expressing *D2A21*. Some necrotic lesions developed when infiltrated at 10^6^ CFU/ml. It was previously reported that transgenic tobacco expressing the AMPs cecropin together with M39 also conferred disease resistance incited by *P*. *syringae* pv. *tabaci*: necrosis was only observed in infiltrated zones with 10^5^ and 10^6^ CFU/ml [36]. The *D2A21* transgenic citrus showed marked resistance to canker caused by *X*. *citri*, when challenged with both leaf infiltration and spray inoculation. However, *D2A21* transgenic citrus appeared not to reduce HLB development. No significant Las titer difference was detected in roots of transgenic plants expressing *D2A21* compared to control plants. There are several possible reasons that D2A21 enhanced disease resistance incited by *P*. *syringae* pv. *tabaci* and *X*. *citri*, but not HLB caused by Las. First, it might be partially due to the fact that D2A21 is less active against Liberibacter (Fig 2) compared with *X*. *citri* [17]. D2A21 as well as other AMPs are presumed to target the cell membranes of microbes resulting in growth inhibition or cell death [9, 10]. It has been shown that D2A21 can alter the plasma membrane of conidia of Ascomycetes [18]. It will be interesting to investigate if Lcr is more resistant to other AMPs and find out the underlying mechanisms of resistance. Second, it is worth noting that *P*. *syringae* pv. *tabaci* and *X*. *citri* are extracellular pathogens and their pathosystems are well-described [37, 38]. Las is an intercellular bacterium limited to the phloem and its pathogenesis remains unclear. It is possible that intercellular bacteria such as Las have less exposure to D2A21 than extracellular pathogens, or the amount of D2A21 protein expressed in the phloem was not sufficient to inhibit Las growth. Our earlier studies showed that the same D35S promoter directed marker gene expression at similar levels in both leaves and phloem [39], suggesting that D2A21 should be present in the phloem. GUS expression driven by the constitutive 35S promoter has been shown to be at similar levels in leaf and root of transgenic Carrizo, in addition two motifs specific for root expression were identified in the 35S promoter [40]. We also observed similar GUS expression in D35S driven transgenic Carrizo leaf and root by staining (Stover, unpublished). In addition, we recently reported that transgenic Carrizo expressing a modified plant thionin driven by the same D35S promotor used in this study significantly reduced canker symptoms and bacterial growth with leaf infiltration and reduced Las titer both in leaf and root [25]. It should be noted that a recent study reported that transgenic citrus expressing synthesized cecropin B driven by a phloem specific promoter reduced susceptibility to HLB [27]. Therefore, it will be interesting to investigate whether D2A21 can reach sufficient level to inhibit Las via overexpression targeted to citrus phloem by a strong phloem promoter we recently identified from citrus (Belknap et al., submitted). Taken together, these results underscore the difficulty in controlling HLB incited by Las.

## Supporting information

S1 Table Las detection from leaves and roots in control plants and transgenic plants of Carrizo expressing *D2A21* nine months after grafting with Las-infected rough lemon. (PDF)

S1 Fig Confirmation of D2A21 insertion in transgenic tobacco plants by PCR. Total DNA from transgenic plants was amplified with primers designed to span from within the D35S promoter through the Nos terminator regions. M: DNA molecular ladder; P: positive control with plasmid carrying D2A21; N: nontransformed control plant; G: negative control with *gus* amplification using pBinARS/Plus-*gus* transgenic plant genomic DNA; Lanes 5–13: independent transgenic tobacco plants carrying *D2A21*.
(TIF)

S2 Fig PCR confirmation of D2A21 insertion in transgenic Carrizo. Total DNA from transgenic plants was amplified with primers designed to span from within the D35S promoter through the Nos terminator regions. M: DNA molecular ladder; P: positive control with plasmid carrying *D2A21*; W: water control; N: nontransformed control plant; Lanes 5–29: independent transgenic Carrizo plants carrying *D2A21*.
(TIF)
