# Supplementary material for: Transgenic expression of antimicrobial peptide D2A21 confers resistance to diseases incited by Pseudomonas syringae pv. tabaci and Xanthomonas citri, but not Candidatus Liberibacter asiaticus
Source: PLoS One. 2017 Oct 19;12(10):e0186810. doi: 10.1371/journal.pone.0186810 (PMC5648250; doi:10.1371/journal.pone.0186810)
Supplement: S1 Table — (PDF) [file pone.0186810.s001.pdf]

Table S1 Las detection from leaves and roots in control plants and transgenic plants of Carrizo expressing *D2A21* nine months after grafting with Las-infected Rough Lemon.

Plant	Old leaf Ct_{Las}	New leaf Ct_{Las}	Root Ct_{Las}
Wildtype Control l	19.70±0.54	25.27±0.12	33.88±1.06
Wildtype Control 2	19.77±0.41	22.94±1.71	31.54±0.20
Wildtype Control 3	17.24±0.92	34.44±0.14	34.69±0.04
Wildtype Control 4	18.79±0.61	26.16±0.85	33.67±0.24
Wildtype Control 5	18.71±0.82	24.40±0.57	31.28±0.13
Wildtype Control 6	21.15±0.98	23.61±2.77	32.85±0.06
D2A21-C16-5	21.01±0.79	ND	31.52±0.19
D2A21-C16-6	20.14±0.72	25.31±0.73	32.39±0.47
D2A21-C16-7	22.79±1.07	ND	33.1±0.10
D2A21-C18-1	19.92±0.68	20.62±0.49	30.51±0.01
D2A21-C18-2	19.29±0.72	20.86±1.67	32.56±0.28
D2A21-C18-3	17.30±0.63	20.14±0.85	30.96±0.10
D2A21-C18-4	23.95±0.94	22.43±0.47	32.32±0.16
D2A21-C18-5	20.42±1.37	22.59±0.70	30.07±0.09
D2A21-C18-6	19.63±0.76	22.21±0.39	32.41±0.03
D2A21-C20-2	20.42±0.28	21.41±1.33	31.76±0.26
D2A21-C20-3	20.98±0.28	21.46±0.66	36.18±1.01
D2A21-C20-4	21.37±0.86	21.26±1.21	32.48±0.13
D2A21-C20-6	17.37±0.93	23.41±0.50	31.30±0.00
D2A21-C20-8	20.00±0.85	22.43±1.24	32.03±0.49
D2A21-C21-3	19.00±0.95	24.89±0.00	33.13±0.10
D2A21-C21-4	22.39±0.80	24.20±0.00	33.10±0.36
D2A21-C21-7	20.24±0.86	30.40±0.03	32.44±0.70
D2A21-C22-1	22.72±0.78	ND	32.95±0.14
D2A21-C22-2	19.88±0.74	28.90±0.02	32.25±0.47
D2A21-C22-6	16.69±0.65	27.16±0.01	30.80±0.07
D2A21-C24-2	16.37±0.30	27.89±0.06	32.53±0.21
D2A21-C24-8	18.44±1.30	ND	32.03±0.30
D2A21-C25-3	21.84±0.01	ND	40.0±0.00
D2A21-C26-2	22.11±1.04	24.11±0.03	30.09±0.30
D2A21-C26-3	37.56±3.45	38.52±0.02	37.99±0.18
D2A21-C26-4	19.75±0.59	26.90±0.01	27.98±0.84
D2A21-C26-6	20.04±0.44	25.88±0.01	31.61±0.50
D2A21-C26-7	19.11±0.47	24.83±0.01	29.05±0.83

ND: No leaves for detection.
The mean threshold cycle values (Ct) with standard deviation from two replicates are listed
