# Supplementary figures and images for: Transgenic expression of antimicrobial peptide D2A21 confers resistance to diseases incited by Pseudomonas syringae pv. tabaci and Xanthomonas citri, but not Candidatus Liberibacter asiaticus
Source: PLoS One. 2017 Oct 19;12(10):e0186810. doi: 10.1371/journal.pone.0186810 (PMC5648250; doi:10.1371/journal.pone.0186810)

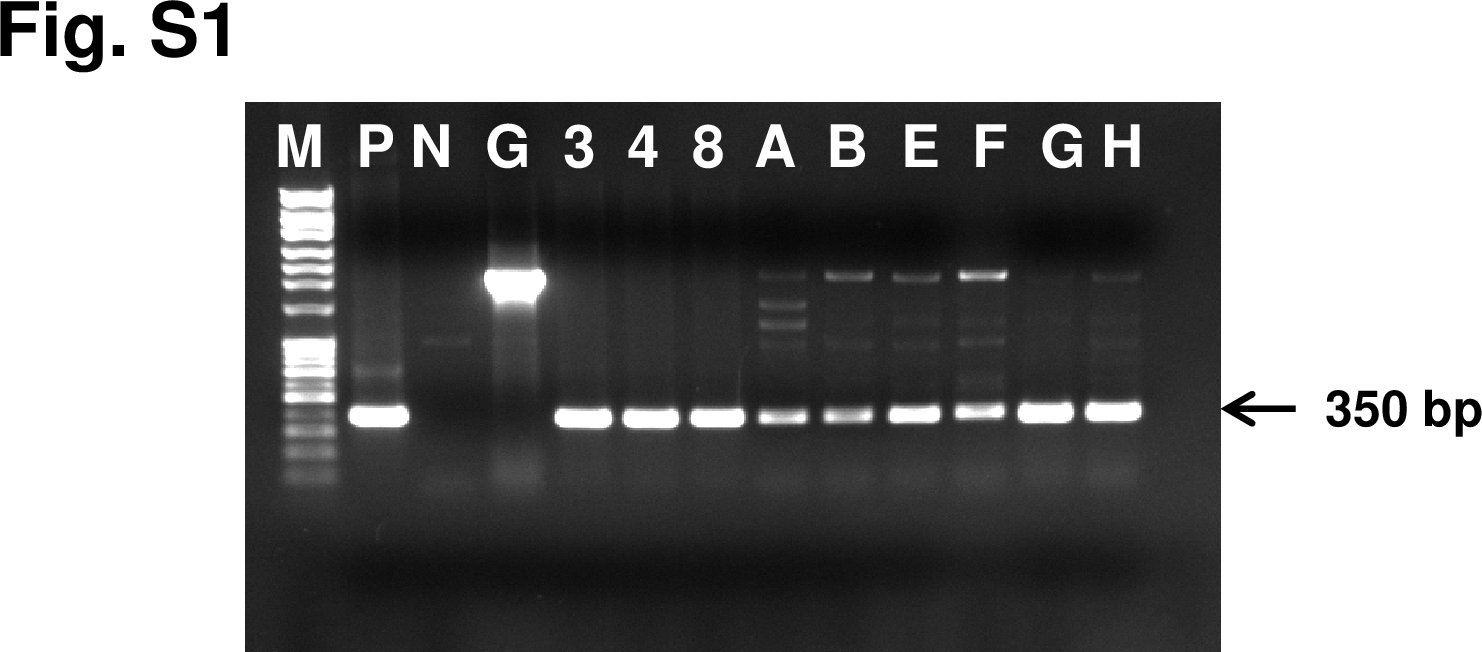

Supplement: S1 Fig — Total DNA from transgenic plants was amplified with primers designed to span from within the D35S promoter through the Nos terminator regions. M: DNA molecular ladder; P: positive control with plasmid carrying D2A21; N: nontransformed control plant; G: negative control with gus amplification using pBinARS/Plus-gus transgenic plant genomic DNA; Lanes 5–13: independent transgenic tobacco plants carrying D2A21. (TIF) [file pone.0186810.s002.tif]

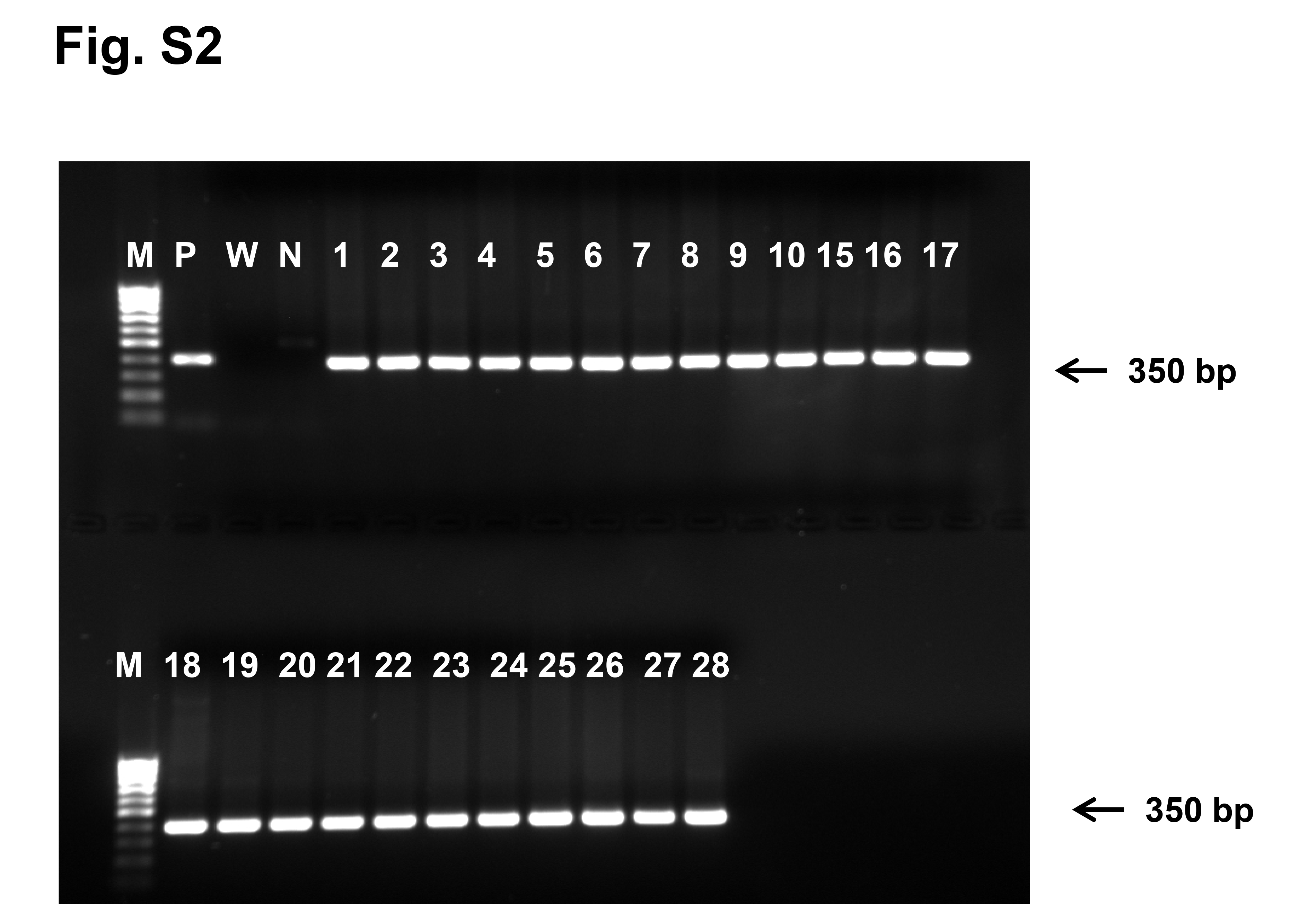

Supplement: S2 Fig — Total DNA from transgenic plants was amplified with primers designed to span from within the D35S promoter through the Nos terminator regions. M: DNA molecular ladder; P: positive control with plasmid carrying D2A21; W: water control; N: nontransformed control plant; Lanes 5–29: independent transgenic Carrizo plants carrying D2A21. (TIF) [file pone.0186810.s003.tif]
